# Selection of reference genes for quantitative real-time RT-PCR assays in different morphological forms of dimorphic zygomycetous fungus *Benjaminiella poitrasii*

**DOI:** 10.1371/journal.pone.0179454

**Published:** 2017-06-09

**Authors:** Ejaj K. Pathan, Vandana Ghormade, Mukund V. Deshpande

**Affiliations:** 1 Biochemical Sciences Division, AcSIR-CSIR-National Chemical Laboratory, Pune, India; 2 Nanobioscience Group, Agharkar Research Institute, Pune, India; Friedrich Schiller University, GERMANY

## Abstract

*Benjaminiella poitrasii*, a dimorphic non-pathogenic zygomycetous fungus, exhibits a morphological yeast (Y) to hypha (H) reversible transition in the vegetative phase, sporangiospores (S) in the asexual phase and zygospores (Z) in the sexual phase. To study the gene expression across these diverse morphological forms, suitable reference genes are required. In the present study, 13 genes *viz*. *ACT*, *18*S r*RNA*, *eEF1α*, *eEF-Tu*,*eIF-1A*, *Tub-α*, *Tub-b*, *Ubc*, *GAPDH*, *Try*, *WS-21*, *NADGDH* and *NADPGDH* were evaluated for their potential as a reference, particularly for studying gene expression during the Y-H reversible transition and also for other asexual and sexual life stages of *B*. *poitrasii*. Analysis of RT-qPCR data using geNorm, normFinder and BestKeeper software revealed that genes such as *Ubc*, *18S rRNA* and *WS-21* were expressed at constant levels in each given subset of RNA samples from all the morphological phases of *B*. *poitrasii*. Therefore, these reference genes can be used to elucidate the role of morpho-genes in *B*. *poitrasii*. Further, use of the two most stably expressed genes (*Ubc* and *WS-21*) to normalize the expression of the ornithine decarboxylase gene (Bp*odc*) in different morphological forms of *B*. *poitrasii*, generated more reliable results, indicating that our selection of reference genes was appropriate.

## Introduction

Fungi display morphological plasticity and differentiate in to vegetative, asexual and sexual forms during their life cycle. RNA quantification is an important tool being used to study the correlation between gene expression and morphological outcome of an organism. Northern blotting, reverse transcriptase PCR (RT-PCR), cDNA microarray and quantitative real-time RT-PCR (RT-qPCR) are commonly used for RNA quantification [1]. Among these methods, RT-qPCR is preferred due to its high sensitivity, robustness, high specificity, good reproducibility and a wide range [2, 3]. However, to get the unbiased results in RT-qPCR, gene expression data must be normalized with appropriate reference gene(s). The number of genes involved in basic cellular functions, such as ribosomal genes (*5*.*8S rRNA*, *18S rRNA*, *28S rRNA*, *WS-21*, *WS-41*) and those encoding actin (*ACT*), β-tubulin (*Tub-b*), translation elongation factor (*eEF1*, *eEF2*, *eEF-Tu*, *eIF-1A*) and glyceraldehyde-3-phosphate dehydrogenase (*GAPDH*) were found to be expressed constitutively in different phases of the life cycle of fungi [1, 4–6]. These genes were commonly used as reference genes in fungi. However, *ACT* and *GAPDH* (named as *TDH3*) were reported to be unsuitable as reference genes in case of *Saccharomyces cerevisiae* [7]. While Yan and Liou [8] observed that in addition to *ACT* and *GAPDH*, translation elongation factors in case of *Phytophthora parasitica* were unsuitable as reference genes. Therefore, it is essential to identify potential reference genes in individual organism being studied for the specific purpose.

*Benjaminiella poitrasii* is a dimorphic, non-pathogenic, zygomycetous fungus being studied extensively to understand biochemical correlates, such as NAD and NADP-dependent glutamate dehydrogenases and ornithine decarboxylase, of morphogenesis [9–14]. Furthermore, yeast-hypha reversible transition was used as target for screening the antifungal drugs [13, 15–17]. This fungus produces yeast and hyphal form cells in the vegetative phase while sporangiospores and zygospores are produced in the asexual and sexual phase, respectively [15]. In the present investigation, 13 candidate genes were evaluated for their potential as reference gene in all the morphological forms of *B*. *poitrasii* for gene expression studies using RT-qPCR. Further, suitability of selected reference genes was confirmed by using them to normalize the expression of ornthine decarboxylase gene (Bp*odc*) in different morphological forms of *B*. *poitrasii*.

## Materials and methods

### Strains and culture conditions

*Benjaminiella poitrasii* NCIM1240 (National collection of Industrial Microorganisms, Pune, India) was maintained on YPG (yeast extract 0.3%, peptone 0.5%, 1% glucose) medium. Initially for RNA isolation, 8 × 10^6^ sporangiospores (S) were inoculated in 50 ml YP medium containing 1% glucose, grown at 37°C for 24 h at 180 rpm and the budding yeast cells (Y) obtained were used as a inoculum. Yeast cells were obtained by inoculating 8 × 10^6^ yeast cells from inoculum per 50 ml of medium into 200 ml of YP medium containing 1% glucose under shaking condition at 180 rpm at 37°C. The yeast cells were harvested after 12 and 24 h. Hyphal cells were grown in the absence of glucose, 8 × 10^6^ yeast cells from the inoculum were inoculated per 50 ml of medium into 200 ml of YP medium under shaking condition (180 rpm) at 28°C. The hyphal cells were harvested after 12 h and 24 h using G1 filter [10]. To obtain yeast and hyphal cells grown in the same medium, 8 × 10^6^ yeast cells were inoculated per 50 ml of medium into 100 ml YP medium containing 0.1% glucose, incubated at 28°C under constant shaking (180 rpm) for 12 h and then separated through G1 filter [10].

### Isolation of sporangiospores and zygospores

The sporangiospores (S) from 5 d, 10 d and 15 d old slants and zygospores (Z) from 7 d, 15 d and 30 d old slants were isolated as described earlier [15]. For instance, the growth on 1% YPG agar slants were lightly scraped to obtain asexual sporangiospores free from yeast, mycelium and zygospores as determined by light microscopy (S1 Fig). For isolation of sexual zygospores, the underlying mycelial bed was scrapped off and crushed with mortar and pestle in distilled water. To obtain zygospores free from other growth forms, the suspension was filtered through muslin cloth and centrifuged at 500g for 15 sec. The washing and centrifuging continued till the clear suspension of zygospores, as seen under light microscope (S1 Fig), was obtained. The sporangiospores and zygospores thus obtained were used for RNA isolation.

### Isolation of RNA

The different morphological form cells were harvested by filtration through Whatmann No. 1 paper, washed with sterile water and ground using mortar and pestle under liquid N_2_. After grinding, 0.3 g powder was transferred to extraction buffer and the total RNA was isolated using RNeasy mini kit according to manufacturer’s instructions (Qiagen, Germany). RNA concentration in each sample was estimated at A_260_ nm using spectrophotometer [18]. The quality of extracted RNA was analyzed on 1.2% agarose gel by electrophoresis.

### Synthesis of cDNA

Total RNA isolated from different morphological forms of *B*. *poitrasii* were treated separately with DNase according to manufacturer’s protocol (Promega, USA), to remove contaminant genomic DNA, if any. The reaction mixture (10 μl) containing 1 μg RNA in 8 μl volume, 1 μl of 10x DNase buffer and 1 μl of RNase free DNase (1U/μl) was incubated at 37°C for 30 min. The reaction was stopped by adding 1 μl of DNase stop solution. The DNase was inactivated by heating the reaction mixture at 65°C for 10 min. The DNase treated RNA(1 μg) from respective samples were used for first strand cDNA synthesis using Omniscript RT-PCR kit (Qiagen, Germany) according to manufacturer’s instructions using oligo(dT)_15_ primer. After 10 x dilution with ddH_2_O, 2 μl of synthesized cDNA was used as a template in RT-qPCR reactions.

### Primers and PCR conditions

The candidate reference genes evaluated in the present study, primer sequences and other details are listed in Table 1. The sequences of *18S rRNA*, *ACT* and eukaryotic translation elongation factor 1α (*eEF1* α) were obtained from NCBI GenBank (http://www.ncbi.nlm.nih.gov/). For other genes the primers were designed from conserved regions of respective genes reported for other fungi in NCBI GenBank database. The primers, Bpodc F (5'- TACGCCATGAAGTGTAACGG-3') and Bpodc R (5'- CTTGCAGGGTTGCGCGTA GA-3'), were used to amplify the ornthine decarboxylase (Bp*odc*) gene fragment. The primer pair amplified a PCR product of 150 bp at 62°C annealing.

**Table 1:** List of the candidate reference genes evaluated in *B*. *poitrasii* and sequences of primers along with optimized annealing temperatures, size of the fragments they amplified and PCR efficiency.

Sr. No.	Gene	Accession No.	Primer Sequences (5'- 3')	Primer Tm (°C)	Size (bp)	PCR Efficiency (%)	Source
1.	***ACT* (β- actin)**	KJ831633	**F** ATGCAGACCGCGGCGCAGAG **R** TTATGAAATGCGATGTGGAT	62.3	165	98.4	[19]
2.	***18SrRNA* (component of 18S ribosome)**	KJ831634	**F** ATTGACAGATTGAAAGCTCT **R** TGGCTTCTTACAGAGACTAT	63.2	150	99.2	[20]
3.	***eEF1α* (translation elongation factor)**	KJ831635	**F** AAGGCTGGTTCCAAGACTGG **R** TGGTCGTCTCTTTCGCTCCT	51.2	210	99.6	[19]
4.	***eEF -Tu* (translation elongation factor—thermo unstable)**	KJ831636	**F** GCGCCGGGCAGCGTGAAAAG R CGGGCGATAACGTGGAAATG	50.5	140	100	Present study
5.	***eIF- 1A* (transcription initiation factor)**	KJ831637	**F** GATTTTCAGGATGAAAAAGC **R** ATGATGTGAGCTTTCTGCGT	52.9	150	100	Present study
6.	***Tub-a* (α-tubulin)**	KJ831638	**F** GAACAGGAAGTGAGCAACGC **R** AACAGCTGATTACCGGCAAA	61.8	240	100	Present study
7.	***Tub-b* (β-tubulin)**	KJ831639	**F** GCGGGCAACAGCTGGGCGAA **R** TGGAACCGTATAACGCGGTG	63.2	170	98.8	Present study
8.	***Ubc* (Ubiquitin-conjugating enzyme)**	KJ831640	**F** AACAAACCGCCGCAGGTGAA **R** ACCGTAAAGAATATGTGCGT	62.5	250	99.2	Present study
9.	***GAPDH* (Glyceraldehyde-3-phosphate dehydrogenase)**	KJ831641	**F** GGCGCGGCGAAAGCGGTGGG **R** TTAAAAAAGCGAGCGAAACC	63.4	200	100	Present study
10.	***Try* (Tryptophan biosynthesis enzyme)**	KJ831642	**F** AACCATACCGGCAGCCATCT **R** CGACCGTGGTGCCGGTGCAG	60.7	160	99.2	Present study
11.	***WS21* (40S ribosomal protein S3A)**	KJ831643	**F** GATCCGACCGGCCGTAACGG **R** ATGAAGCGGCGGTGGAAGCG	62.8	180	100	Present study
12.	***NADPGDH* (NADP-dependent glutamate dehydrogenase)**	KJ843147	**F** CGGGTCTCATGATGGGCGGT **R** TGGCTTCGGGACGGATGTGA	60.0	140	98.4	Present study
13.	***NADGDH* ((NAD-dependent glutamate dehydrogenase)**	KJ843146	**F** GGCTCTGGCGTCCTTTACGA **R** CGGCCTTCTCTGTCAGATCC	58.7	150	98.2	Present study

The amplified PCR products for each primer pair were analyzed on 2% agarose gel to check the specificity of primer pairs.

### Molecular cloning and DNA sequence analysis

The PCR products were cleaned using QIAquick PCR purification system (Qiagen), and cloned into pGEM-Teasy vector system (Promega, USA) and transformed to *Escherichia coli* JM109 competent cells. From these, 20 clones of each gene were selected, plasmid was purified and further characterized by DNA sequencing. Plasmids were sequenced using the Big Dye Kit (Perkin-Elmer, Worthington, UK) and run on an ABI 377automated sequencer from Perkin-Elmer. DNA and deduced amino acid sequences were analyzed using genetics Computer Group package (Madison, WI, USA). The nucleotide and deduced amino acid sequences were identified using the BLAST search at NCBI.

### RT-qPCR analysis

All RT-qPCR reactions were carried out in Eppendorf Mastercycler ep realplex^4^S.

After 10 x dilution with sterile ddH_2_O, 2 μl of cDNA was used as template. The RT-qPCR was performed in a volume of 20 μl containing 8 μl of 2.5 x RealMasterMix (5Prime, Germany); 1 μl of 20 x SYBR Green and 300 nmoles of each primers. The PCR conditions were: 95°C for 1 min, followed by 40 cycles of 95°C/20 s, annealing (at optimum temperature, Table 1)/30 s and 72°C/30 s. A melting curve was performed at the end of each RT-qPCR run by increasing the temperature in a stepwise manner by 0.5°C every 5 s, from 60°C to 95°C. For each primer pair, reaction without template cDNA was served as a negative control, termed as No Template Control (NTC). All the RT-qPCR reactions were performed in triplicate with RNA from two different sets of samples.

### Statistical analysis

The results obtained in the form of Ct values from RT-qPCR experiments were analyzed using the geNorm program (http://medgen.ugent.be/~jvdesomp/genorm) and the gene expression stability (*M*) value was calculated [21]. The optimum number of reference genes required for reliable normalization was obtained by pair-wise variation analysis [21]. For validation, the data was also analyzed with normFinder and BestKeeper software's [22, 23]. Each of these software package uses slightly different parameters to evaluate the stability of reference genes. For instance, in geNorm analysis, the test genes were compared pair-wise and then ranked based on the similarity of their expression profile so that genes with the most stable expression level in a given set of RNA samples could be identified [21]. The other software program, BestKeeper, is based on Ct value correlation and regression analysis. BestKeeper computes the best reference gene(s) based on their geometric mean called as BestKeeper index [23]. On the other hand, normFinder is a model based approach that uses the absolute value of mean plus standard deviation as stability measure. Stability value reflects the combined effect of intra- as well as inter- group variations [22].

The selected reference genes were used to normalize the expression of Bp*odc* gene in different morphological forms of *B*. *poitrasii* by using excel based application for group wise comparison. The gene showing least stable expression was also used to normalize the Bp*odc* gene expression.

The efficiency (E) of a RT-qPCR assay for each of the studied gene was determined by classical calibration dilution curve and slope calculation. From the standard curves, the efficiency was calculated using formula E = [(10^(-1/slope)^ - 1) x 100].

### Estimation of adenosine 3', 5'-cyclic monophosphate (cAMP)

Cyclic AMP content of each sample was measured by using the direct cAMP enzyme immunoassay 96 well kit (Sigma-Aldrich, USA). Yeast cells, hyphae, sporangiospores and zygospores, isolated as mentioned above, were frozen in liquid nitrogen and ground to fine powder. The powder was dissolved in buffer and centrifuged (13000 x g) at 4°C for 15 min. The supernatant thus obtained was used for cAMP estimation according to the manufacturer’s instructions (Sigma-Aldrich, USA).

### Estimation of nicotinamide adenine dinucleotide (phosphate) reduced [NAD(P)H]

The NAD(P)H content was estimated by directly measuring the absorbance of each sample at 340 nm [24]. The frozen cell sample (0.3 g) in liquid nitrogen was ground to fine powder. The sample was dissolved in 3 ml of 100 mM potassium phosphate buffer pH 8.0. The supernatant was obtained by centrifugation at 4°C (13000 x g, 15 min) and the absorbance was measured at 340 nm. The standard calibration curve of NAD(P)H was made (2–200 μg/ml) and concentration of NAD(P)H in test samples were determined by interpolation from the standard calibration curve.

### Estimation of intracellular protein

For estimating the total intracellular proteins in different morphological forms of *B*. *poitrasii*, cells were harvested by filtration and washed with the sterile distilled water and then with 100 mM potassium phosphate buffer (pH 8.0). The Cells (1 g wt. weight) were homogenized in Braun’s homogenizer (Braun, Germany) for 60 s (4 cycles, 15 s each) in the presence of 100 mM potassium phosphate buffer containing 1 mM ethylene diaminetetraaceticacid (EDTA), 1 mM phenylmethylsulfonyl fluoride (PMSF) and 3 mM dithiothreitol (DTT), pH 8.0 (2.5 ml/g of cells). The homogenate was then centrifuged at 12,500 x g for 20 min. The supernatant thus obtained was used to estimate total intracellular protein content by Bradford method using Bovine serum albumin as standard [25].

## Results

### Selection of *B*. *poitrasii* morphological forms of different ages for RT-qPCR studies based on intracellular protein, cAMP and NAD(P)H contents

The intracellular protein contents in yeast form cells of *B*. *poitrasii* were 2 fold more than hyphal cells grown in the same medium (0.1% YPG, 28°C, 12 h) (Table 2). Similarly cAMP and NAD(P)H concentration on equal weight basis were also higher in yeast cells than hyphae. The glucose (1%) in the medium decreased the concentrations of cAMP and NAD(P)H in 12 h grown cells. In sporangiospores and zygospores, the protein, cAMP and NAD(P)H contents decreased as the age increased (Table 2). As the differences in the concentrations of protein, cAMP and NAD(P)H were significant in different morphological forms of different ages, the corresponding samples were used for the analysis of candidate reference genes (S1 Fig).

**Table 2:** Protein, cAMP, and NAD(P)H contents of different morphological forms of *Benjaminiella poitrasii*.

Stage	Sample	Protein# (mg/g of cells wt. weight)	cAMP* (pmol/g of cells wt. weight)	NAD(P)H** (nmol/g of cells wt. weight)
Vegetative	Yeast 12 h	8.5	3000	4394
	Yeast 24 h	8.7	1840	7083
	Hyphae 12 h	4.2	1600	3539
	Hyphae 24 h	5.0	1140	3103
	Yeast 12h 0.1% YPG, 28°C	8.6	3800	4859
	Hyphae 12h 0.1% YPG, 28°C	4.3	1640	3111
Asexual	Sporangiospores 5 d	2.8	1340	3342
	Sporangiospores 10 d	2.0	700	2935
	Sporangiospores 15 d	1.8	540	1748
Sexual	Zygospores 7 d	5.6	1120	3321
	Zygospores 15 d	2.8	900	2648
	Zygospores 30 d	2.3	800	2524

*cAMP content was estimated by using cAMP enzyme immunoassay 96 well kit from Sigma (Product number CA-201).

** NAD(P)H content was estimated by measuring the absorbance of each sample at 340nm and concentration of NAD(P)H was then determined by interpolation from standard calibration curve.

# Protein was estimated by Bradford method using Bovine serum albumin as standard. 0.1% YPG (yeast extract 0.3%, peptone 0.5%, glucose 0.1%)

### Selection of candidate reference genes

The short fragments (140–250 bp) of 10 genes, except *ACT*, *eEF1α* and *18S* rRNA, were amplified, purified, cloned and analyzed in the present study and the sequence data are deposited in the NCBI GenBank database (Table 1).

**Fig 1:**
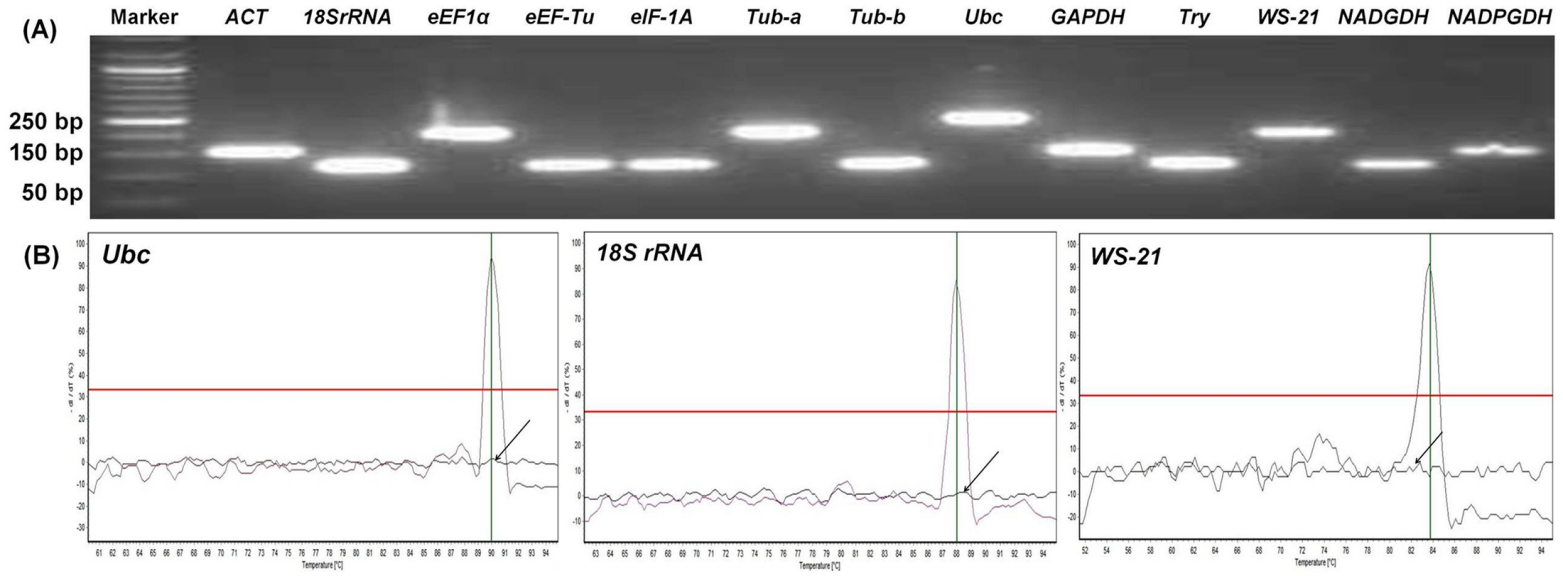
Specificity of primers used for RT-qPCR analysis in *B*. *poitrasii*. **A)** Agarose gel electrophoresis and analysis of amplified products obtained from RT-qPCR. The electrophoresis was done on 2% agarose gel. The names of candidate reference genes are mentioned on top. The figures on left indicate the size of PCR amplicons in base pairs. B) Melting curves indicating single peaks for three representative genes. Arrow indicates the melting curve for the RT-qPCR reaction without template.

The presence of a single amplicon in RT-qPCR for all 13 genes was confirmed by the sharp single peak in melting curve analysis (Fig 1; S2 Fig) Furthermore, the electrophoresis analysis on 2% agarose gel displayed a single sharp band of expected size for each primer set, indicating the specificity of the respective primer in RT-qPCR (Fig 1). The PCR efficiency for all the primer pairs was found to be >98% (Table 1).

### The expression profile of candidate reference genes

**Fig 2:**
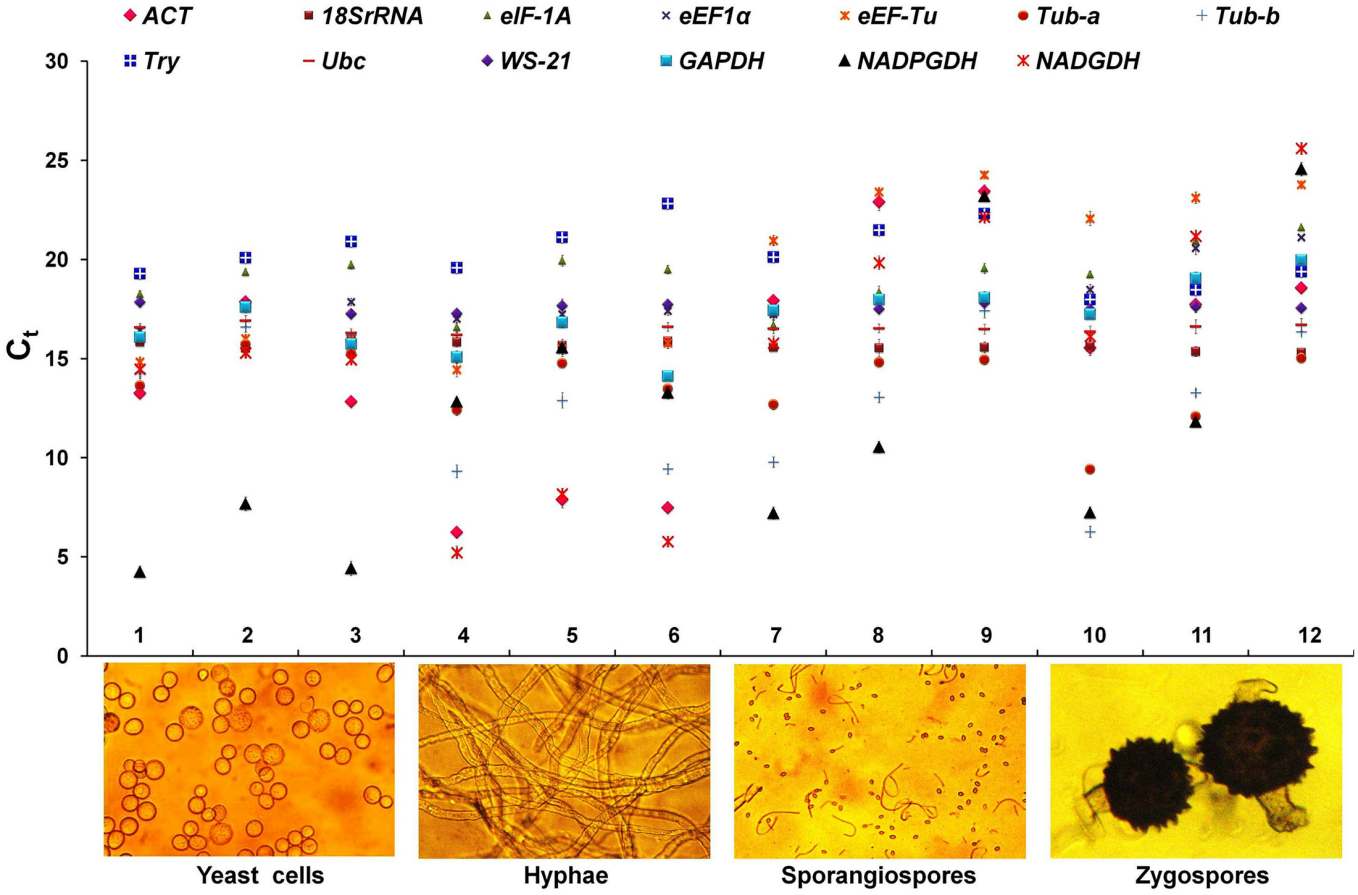
Distribution overview of expression of each candidate reference gene in absolute Ct values for RNA samples of different morphological forms in *B*. *poitrasii*. Samples 1, yeast cells grown in YPG (1%) medium for 12 h; Sample 2, yeast cells grown in YPG medium for 24h; Sample 3, yeast cells grown in YPG (0.1%) medium for 12 h; Sample 4, hyphae grown in YP medium for 12 h; Sample 5, hyphae grown in YP medium for 24 h; Sample 6, hyphae grown in YPG(0.1%) for 12 h.; Sample 7, 5 d old sporangiospores; Sample 8, 10 d old sporangiospores; Sample 9, 15 d old sporangiospores; Sample 10, 7 d old zygospores; Sample 11, 15 d old zygospores; Sample 12, 30 d old zygospores.

The total RNA was isolated from selected samples as mentioned in Table 2 and analyzed by RT-qPCR. The expression profile of *Ubc* (Ct 16.24–17.12), *18S rRNA* (Ct 15.31–15.76) and *WS-21* (Ct 17.42–17.90) showed the minimum variation, as reflected from their Ct values, in all the morphological forms (Fig 2). By contrast, significant variations were observed in expression profile of *ACT* (Ct 6.24–25.77), *Tub-a* (Ct 9.43–15.72), *Tub-b* (Ct 6.27–17.43), *NADGDH* (Ct 5.23–22.15) and *NADPGDH* (Ct 4.25–23.20) genes. If the different life stages *viz*. vegetative, asexual and sexual, were considered then these variations were slightly different. During vegetative stage, in addition to *Ubc*, *18S rRNA* and *WS-21* genes, the expression profile of *eEF1α* (Ct 16.44–17.87) and *eEF-Tu* (Ct 14.44–16.12) showed the least variation. Whereas, the expression profile of *NADGDH* (Ct 5.23–15.29), *NADPGDH* (Ct 4.25–15.56), *Tub-b* (Ct 9.32–16.61) and *ACT* (Ct 6.24–17.86) genes showed the wide variation during vegetative stage (Fig 2). During asexual and sexual stages, in addition to *Ubc*, *18S rRNA* and *WS-21* genes, *GAPDH* (Ct 17.24–19.97) showed the stable expression pattern with minimum variations in Ct values. On the contrary, expressions of *Tub-b* (Ct 6.27–17.43), *NADGDH* (Ct 15.78–25.61) and *NADPGDH* (Ct 7.21–24.56) showed significant variation during asexual and sexual stages of *B*. *poitrasii*. Overall, the stage specific variations were minimum or absent in case of *Ubc*, *18S rRNA* and *WS-21*, indicated their potential as a suitable reference genes (Fig 2; S3 Fig).

### Analysis of expression stability of candidate reference genes

**Fig 3:**
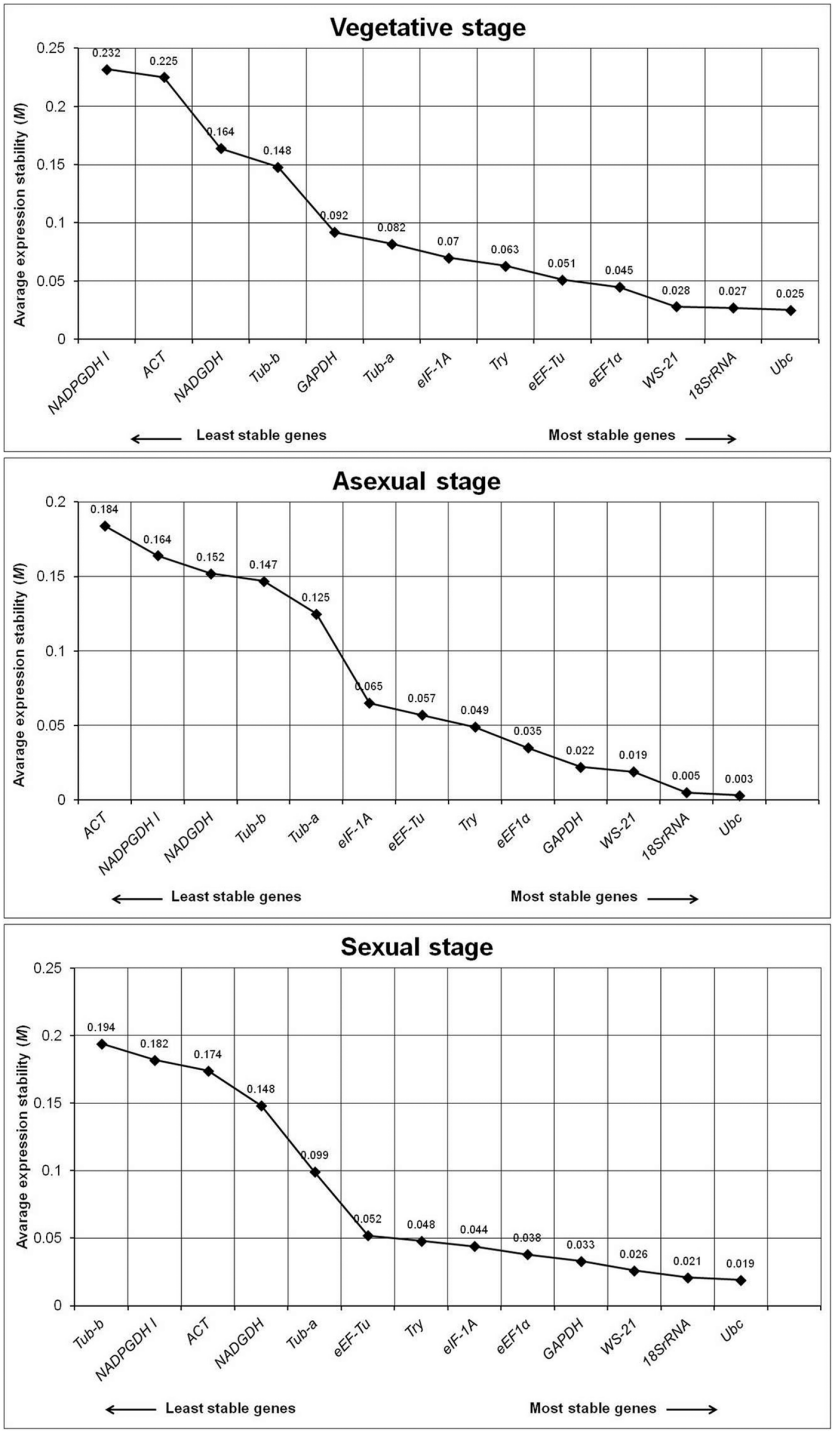
geNorm—based ranking of the candidate reference genes during different morphological stages of *B*. *poitrasii*. Genes were ranked from the least stable (on the left) to the most stable (on the right) according to their *M* value (Y axis). This classification was independently performed by using different sets of conditions. Vegetative stage, 6 samples of yeast and hyphal cells; Asexual stage, 3 samples of sporangiospores; Sexual stage, 3 samples of zygospores as described in Fig 2 legend.

The gene expression stability of each gene was indicated by their *M* values and calculated by using geNorm program. The genes with the lowest *M* values were considered as most stable. The 13 candidate reference genes used in the present study were ranked based on their *M* values (Fig 3). For vegetative stage, the expression of *Ubc* was most stable followed *18S rRNA* and *WS-21*. The expression of *eEF1α* was also found to be stable during vegetative stage. However, the expression of *NADGDH*, *NADPGDH*, *Tub-a*, *Tub-b* and *ACT* were found to vary as evident from their high ‘*M*’ values. For asexual as well as sexual stages, the expression of *Ubc* was most stable followed by *18S rRNA* and *WS-21*. The expression stabilities of reference genes were also evaluated with two other software packages viz., normFinder and BestKeeper (Table 3;S1–S3 Tables). The stability rankings of reference genes calculated by geNorm and normFinder were close, whereas stability ranking by BestKeeper was slightly different. Moreover, *Ubc*, *18S rRNA* and *WS-21* were found to be the most stable genes in consensus with all 3 software packages (Table 4).

**Table 3:** Ranking of thirteen candidate reference gene in order of their expression stability as calculated by normFinder program.

Rank	Vegetative stage		Asexual stage		Sexual stage	
	Gene	Stability	Gene	Stability	Gene	Stability
**1**	***Ubc***	0.063	***Ubc***	0.006	***Ubc***	0.007
**2**	***18SrRNA***	0.069	***18SrRNA***	0.007	***18SrRNA***	0.008
**3**	***WS-21***	0.071	***WS-21***	0.009	***WS-21***	0.009
**4**	*eEF1α*	0.082	*GAPDH*	0.013	*GAPDH*	0.012
**5**	*eEF-Tu*	0.086	*eEF1α*	0.016	*eEF1α*	0.015
**6**	*Try*	0.096	*Try*	0.020	*eIF-1A*	0.017
**7**	*eIF-1A*	0.105	*eEF-Tu*	0.023	*Try*	0.037
**8**	*Tub-a*	0.126	*eIF-1A*	0.026	*eEF-Tu*	0.044
**9**	*GAPDH*	0.130	*Tub-a*	0.130	*Tub-a*	0.129
**10**	*Tub-b*	0.198	*Tub-b*	0.203	*NADGDH*	0.154
**11**	*NADGDH*	0.212	*NADGDH*	0.224	*ACT*	0.193
**12**	*ACT*	0.371	*NADPGDH*	0.238	*NADPGDH*	0.246
**13**	*NADPGDH*	0.394	*ACT*	0.270	*Tub-b*	0.393

**Table 4:** Comparison of expression stability of reference genes in different phases of *Benjaminiella poitrasii* as determined by geNorm, BestKeeper and normFinder software packages.

Rank	Vegetative stage			Asexual stage			Sexual stage		
	geNorm	Best Keeper	norm Finder	geNorm	Best Keeper	norm Finder	geNorm	Best Keeper	norm Finder
1	***Ubc***	***Ubc***	***Ubc***	***Ubc***	***Ubc***	***Ubc***	***Ubc***	***Ubc***	***Ubc***
2	***18SrRNA***	***18srRNA***	***18SrRNA***	***18SrRNA***	***18SrRNA***	***18SrRNA***	***18SrRNA***	***18SrRNA***	***18SrRNA***
3	***WS-21***	***WS-21***	***WS-21***	***WS-21***	***WS-21***	***WS-21***	***WS-21***	***WS-21***	***WS-21***
4	*eEF1α*	*eIF-1A*	*eEF1α*	*GAPDH*	*eEF1α*	*GAPDH*	*GAPDH*	*eIF1A*	*GAPDH*
5	*eEF-Tu*	*eEF1α*	*eEF-Tu*	*eEF1α*	*GAPDH*	*eEF1α*	*eEF1α*	*Try*	*eEF1α*
6	*Try*	*Try*	*Try*	*Try*	*Try*	*Try*	*eIF-1A*	*eEF1α*	*eIF-1A*
7	*eIF-1A*	*GAPDH*	*eIF-1A*	*eEF-Tu*	*Tub-a*	*eEF-Tu*	*Try*	*eEF-Tu*	*Try*
8	*Tub-a*	*eEF-Tu*	*Tub-a*	*eIF1A*	*eEF-Tu*	*eIF1A*	*eEF-Tu*	*ACT*	*eEF-Tu*
9	*GAPDH*	*Tub-a*	*GAPDH*	*Tub-a*	*eIF1A*	*Tub-a*	*Tub-a*	*GAPDH*	*Tub-a*
10	*Tub-b*	*Tub-b*	*Tub-b*	*Tub-b*	*NADPGDH*	*Tub-b*	*NADGDH*	*NADPGDH*	*NADGDH*
11	*NADGDH*	*NADPGDH*	*NADGDH*	*NADGDH*	*NADGDH*	*NADGDH*	*ACT*	*NADGDH*	*ACT*
12	*ACT*	*NADGDH*	*ACT*	*NADPGDH*	*ACT*	*NADPGDH*	*NADPGDH*	*Tub-a*	*NADPGDH*
13	*NADPGDH*	*ACT*	*NADPGDH*	*ACT*	*Tub-b*	*ACT*	*Tub-b*	*Tub-b*	*Tub-b*

### Optimum number of reference genes for normalization

The minimal number of reference genes required for reliable normalization was determined by calculating the pair-wise variation (V_n/n+1_) between two sequential normalization factors (NFn and NFn+1) using geNorm program. The cutoff threshold was set at V = 0.15, below which the inclusion of additional reference gene is not required.

**Fig 4:**
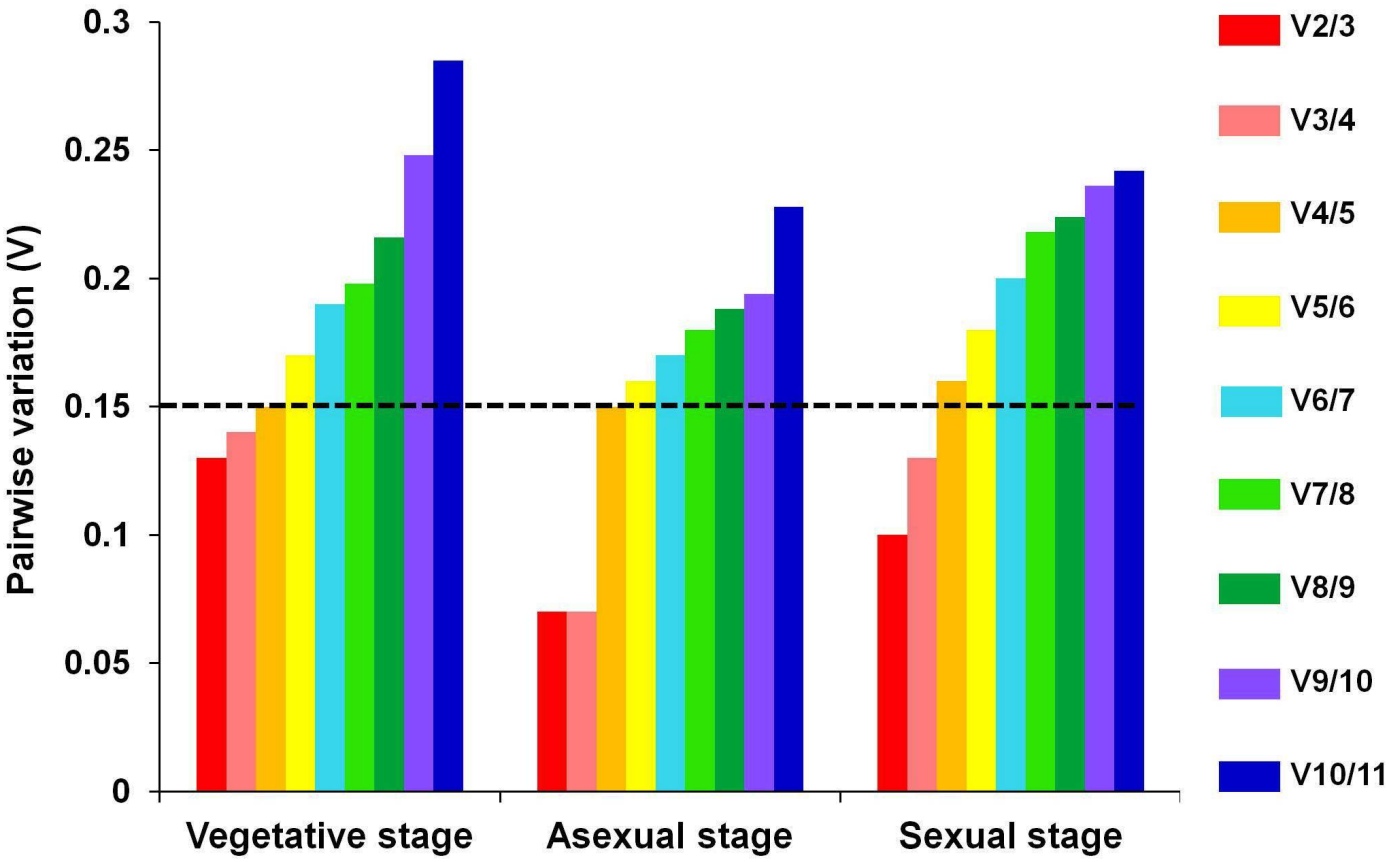
Pairwise variation analysis between NFn and NFn+1 to determine the optimal number of genes for reliable normalization. Values below the 0.15 threshold mean that *n* genes might be sufficient.

As shown in Fig 4, for the pool of “vegetative stage” i.e. during Y-H dimorphism, pair-wise variation analysis indicated that two most stably expressed genes namely *Ubc* in combination with *18S rRNA* or *WS-21* (V_2/3_ = 0.13) could be used for normalization to obtain the reliable data. For the gene expression studies during asexual and sexual stages, combination of *Ubc* and *18S rRNA*, was found to be sufficient with V_2/3_ of 0.07 and 0.1 respectively, for RT-qPCR data normalization. Both of these values were below the cut-off threshold of 0.15. Therefore, use of two most stably expressed genes would be sufficient as reference genes for reliable data normalization (Fig 4).

### Analysis of Bp*odc* gene expression during Y-H dimorphism and at different life stages of *B*. *poitrasii*

**Fig 5:**
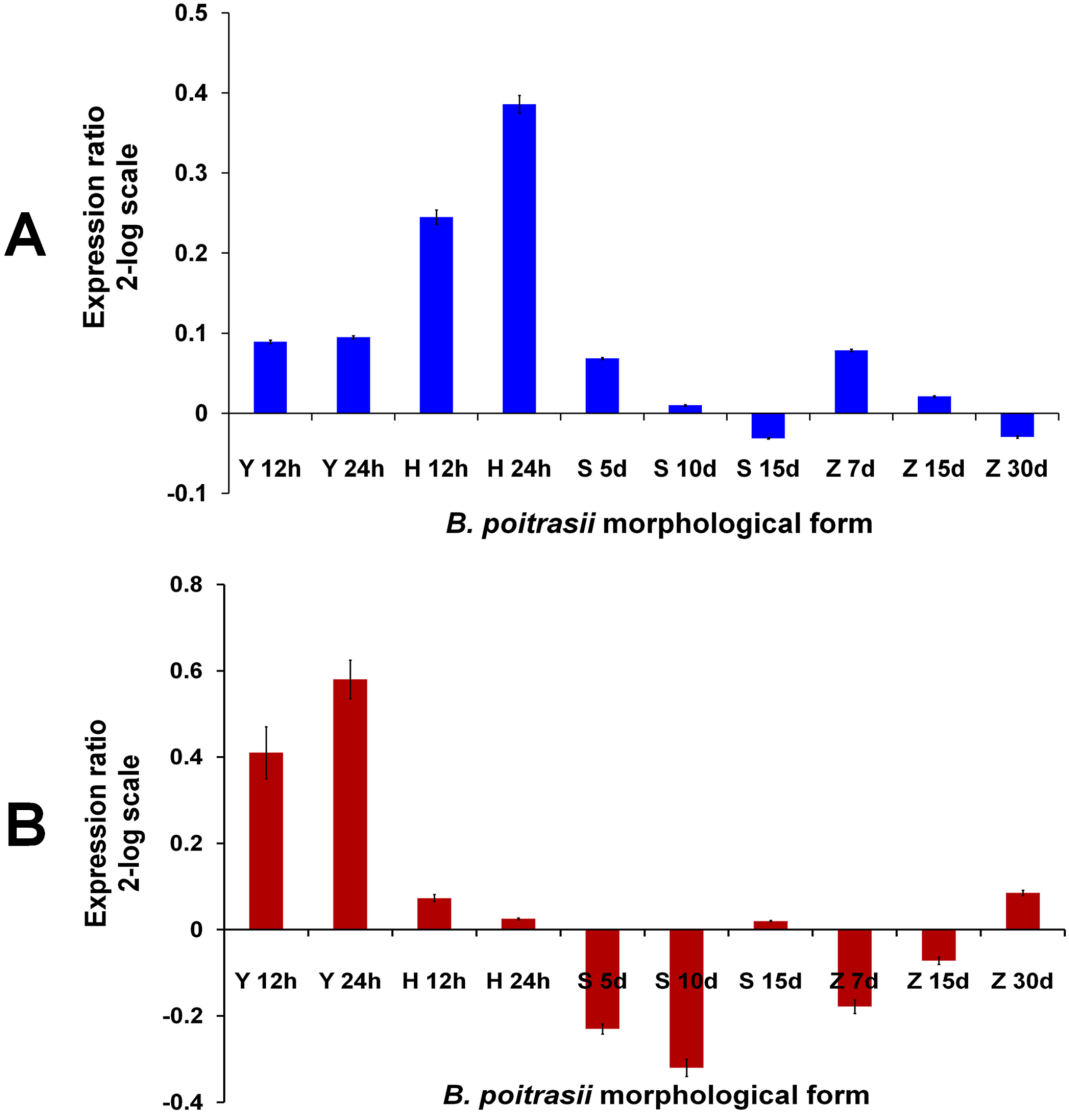
Quantification of ornithine decarboxylase (Bp*odc*) gene transcripts by RT-qPCR in different morphological forms of *B*. *poitrasii*. The amount of Bp*odc* transcripts present in different morphological forms of *B*. *poitrasii* (Y, yeast cells; H, hyphae; S, sporangiospores; Z, zygospores) at different time points (h, hours; d, days) was measured and normalized with the transcripts from *Ubc* and *WS-21* (A) and *Tub-b* (B) as reference.

Ornithine decarboxylase *(odc)* gene and accordingly enzyme activity were found to be differentially expressed during the morphological transition in fungi. The expression is usually higher in hyphae than in yeast cells [14, 26–27]. Therefore, to validate that we have made the correct choice of reference genes, RT-qPCR analysis was performed for Bp*odc*, *Ubc*, *WS-21* and *Tub-b*. Data obtained for Bp*odc* was then normalized with *Ubc* and *WS-21* (Fig 5A) as suggested by our results and with *Tub-b* (Fig 5B) independently. As shown in Fig 5A, with *Ubc* and *WS-21* as reference genes, the expression Bp*odc* was found to be higher in hyphae than in yeast cells, sporangiospores and zygospores. The highest expression was seen in 24h grown hyphae, whereas the lowest expression was observed in 15d old sporangiospores and 30d old zygospores.

In contrast, when normalization was performed using *Tub-b* as reference (Fig 5B), the Bp*odc* was found to be repressed in hypha as compare to yeast cells. Further, the expression was found to be continuously decreased in 5d and 10d old sporangiospores and 7d and 15d old zygospores, which was suddenly increased in 15d old sporangiospores and 30d old zygospores. This data gave an unexpected expression pattern of Bp*odc* gene due to normalization with wrong reference gene. It is thus indicating that the normalization with inappropriate reference gene(s) would result in completely different conclusion (Fig 5B).

## Discussion

To understand the molecular mechanisms involved in fungal morphogenesis, expression of the key genes involved in this process must be analyzed. RT-qPCR is a method of choice for the study of gene expression, provided that data is normalized with appropriate reference genes. For instance, in case of *Aspergillus niger*, *ACT*, *SarA* (alfa-sarcin) and *Cox5* (cytochrome c oxidase subunit V) were identified as reference genes to study the gene expression under different cultivation conditions [5]. Fang and Bidochka [6] suggested the use of six reference genes viz., *ACT*, *GAPDH*, *18S rRNA*, *Tef* (translation elongation factor), *Try* and *Ubc* to understand the molecular mechanism of conidiogenesis and insect pathogenesis by *Metarhizium anisopliae*. In a cereal pathogen *Fusarium graminearum*, *eEF1a* and *Ubc* were selected as reference genes to study the gene expression during vegetative growth, ascospore formation and trichothecene production [28]. In *S*. *cerevisiae*, reference genes were identified for growth phase-related mRNA profiling. For this, the samples of *S*. *cerevisiae* grown in different physiological states during long term cultures, grown on different carbon sources and genetic contexts were analyzed. The use of combination of any 3 genes among *ALG9* (mannosyl transferase), *TAF10* (RNA pol. II transcription factor), *TFC I* (RNA pol. III transcription factor) and *UBC6* (ubiquitin protein ligase) was recommended for data normalization. While, the genes such as *ACT1*, *PDA1* (pyruvate dehydrogenase) and *TDH3* (glyceraldehyde-3-phosphate dehydrogenase) were found to be unsuitable as reference genes [7]. Li et al. [4] recommended the use of *RDN5*.*8* (coding for 5.8S rRNA), *UBC13* (ubiquitin C) and *PGK1* (phosphoglycerate kinase) alone or in combination as reference genes for analyzing the differences in gene expression levels in *Candida glabrata* during azole treatment. To analyze the changes in gene expression levels between *Candida albicans* biofilm cells and planktonic cells, Nailis et al. [1] recommended the use of five reference genes viz. *ACT1*, *PMA1* (plasma membrane ATPase pump), *RIP* (ubiquinol cytochrome-c reductase complex component), *RPP2B* (cytosolic ribosomal acidic protein P2B) and *LSC2* (succinyl-CoA synthetase β-subunit fragment) for accurate data normalization. Recently, Llanos et al. [29] proposed the use of any 3 genes out of *UbcB*, *Sac7* (Rho GTPase activator), *Fis1* (mitochondrial membrane fission protein), *SarA*, *TFC1* and *UBC6*, as reference in any field of fungal biology. In case of plant pathogenic fungus *P*. *parasitica*, *Ubc* and *WS-21* were found to be suitable as reference genes and use of *Tub-b* was also recommended for normalization [8]. However, there were only few reports that describe the genes with stable expression during Y-H dimorphism [30]. In case of dimorphic *Mucor circinelloides*, Valle-Maldonado et al. [30] studied 7 genes viz. *28S rRNA*, *eIF-1A* (termed as *tfc-1*), *eEF1α* (termed as *ef-1*), *vma-1* (vacuolar H (+)-ATPase), *tfb4* (component of RNA Pol. II preinitiation complex), *18S rRNA* and *e1* (ubiquitin-activating enzyme) for their potential as reference genes. Among these, they identified *eIF-1A* and *eEf1α* as reference genes to study the gene expression during Y-H morphogenesis. Though ribosomal genes were found to be stably expressed, due to their high abundance were not suggested to be useful as reference genes. In the present investigation, though *B*. *poitrasii* is a dimorphic zygomycete similar to *M*. *circinelloides*, *Ubc* and *WS-21* were found to be more suitable as reference genes than e*EF1α* and *eIF-1A*, to study the gene expression during Y-H transition as well as in asexual and sexual stages of life cycle. This could be ascribed to the wider panel of candidate reference genes and the number of samples analyzed in the present study. The expression of genes such as *ACT*, *Tub-b* and *GAPDH*, which are commonly used as a reference genes for the study of gene expression in fungi [5, 6], varied greatly in different morphological forms of *B*. *poitrasii*. According to the geNorm, within the pool of ‘vegetative stages’ ‘asexual cycle' and ‘sexual cycle’, use of *Ubc* in combination with either *18S rRNA* or *WS-21* was sufficient for data normalization. It can be attributed to the similarity in transcription profiles of *18S rRNA* and *WS-21* as evident from their *M* values.

The importance of using a suitable reference gene for data normalization can be envisaged from the analysis of Bp*odc* gene expression. Normalization with the two most stably expressed genes identified for all stages, namely *Ubc* and *WS21*, clearly demonstrated that the expression of Bp*odc* was lower in yeast cells, sporangiospores and zygospores as compared to the expression level obtained in hyphae, which is in accordance with the previous reports [14, 26–27]. However, normalization with *Tub-b* led to misinterpretation of the results and even a different conclusion.

*B*. *poitrasii* has been extensively studied to understand the biochemical mechanism of dimorphic behavior and also used as a model to screen the inhibitors of Y-H transition [10–13, 15–17]. The relative proportion of active NAD- and NADP-GDH enzymes was reported as biochemical correlate of Y-H dimorphism in *B*. *poitrasii* [10, 11]. Now in the light of identified reference genes, *Ubc* and *WS-21*, it would be useful to know the transcriptional changes in biochemical correlates during Y-H dimorphism in *B*. *poitrasii*.

## Supporting information

S1 Fig Different life forms of *Benjaminiella poitrasii*: a. Yeast cells; b. germinating yeast cells; c. hyphae; d. budding hyphae; e. asexual sporangiospores and f. sexual zygospores. All images were captured at 400X magnification.
(TIF)

S2 Fig The melting curve of candidate reference genes analyzed by RT-qPCR. A single sharp peak was observed for each gene product. The melting curve for no template control (NTC) is indicated by arrow.
(TIF)

S3 Fig Expression stability of candidate reference genes in different life forms of *Benjaminiella poitrasii* by Box-Plot method. (TIF)

S1 Table Analysis of CP data of candidate reference genes by BestKeeper s/w: during vegetative stage (Yeast-hypha dimorphism). (DOCX)

S2 Table Analysis of CP data of candidate reference genes by BestKeeper s/w: during asexual stage (Sporangiospores formation). (DOCX)

S3 Table Analysis of CP data of candidate reference genes by BestKeeper s/w: during sexual stage (Zygospores formation). (DOCX)
