# Supplementary figures and images for: Selection of reference genes for quantitative real-time RT-PCR assays in different morphological forms of dimorphic zygomycetous fungus Benjaminiella poitrasii
Source: PLoS One. 2017 Jun 9;12(6):e0179454. doi: 10.1371/journal.pone.0179454 (PMC5466344; doi:10.1371/journal.pone.0179454)

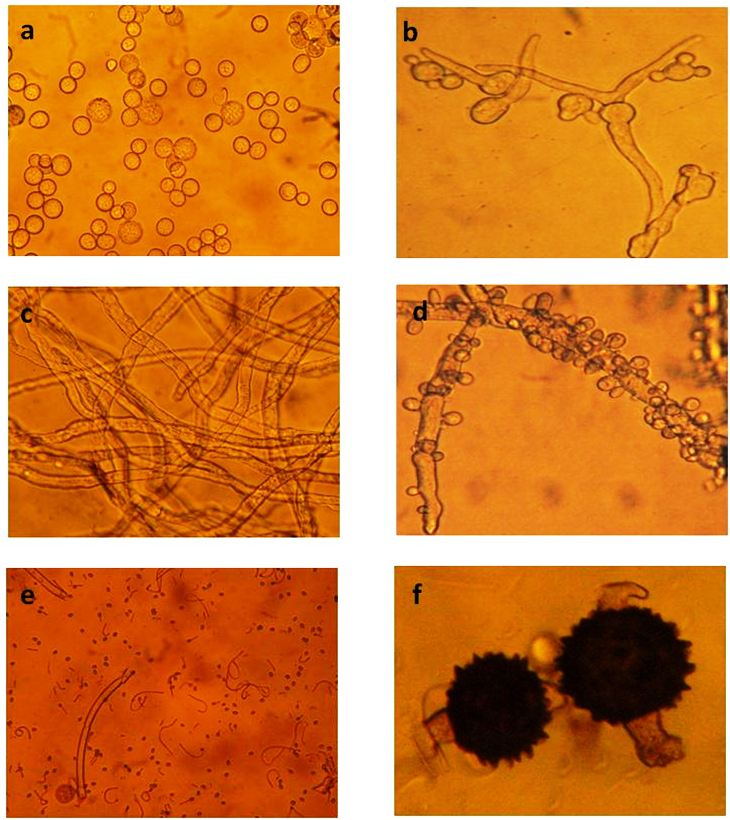

Supplement: S1 Fig — All images were captured at 400X magnification. (TIF) [file pone.0179454.s001.tif]

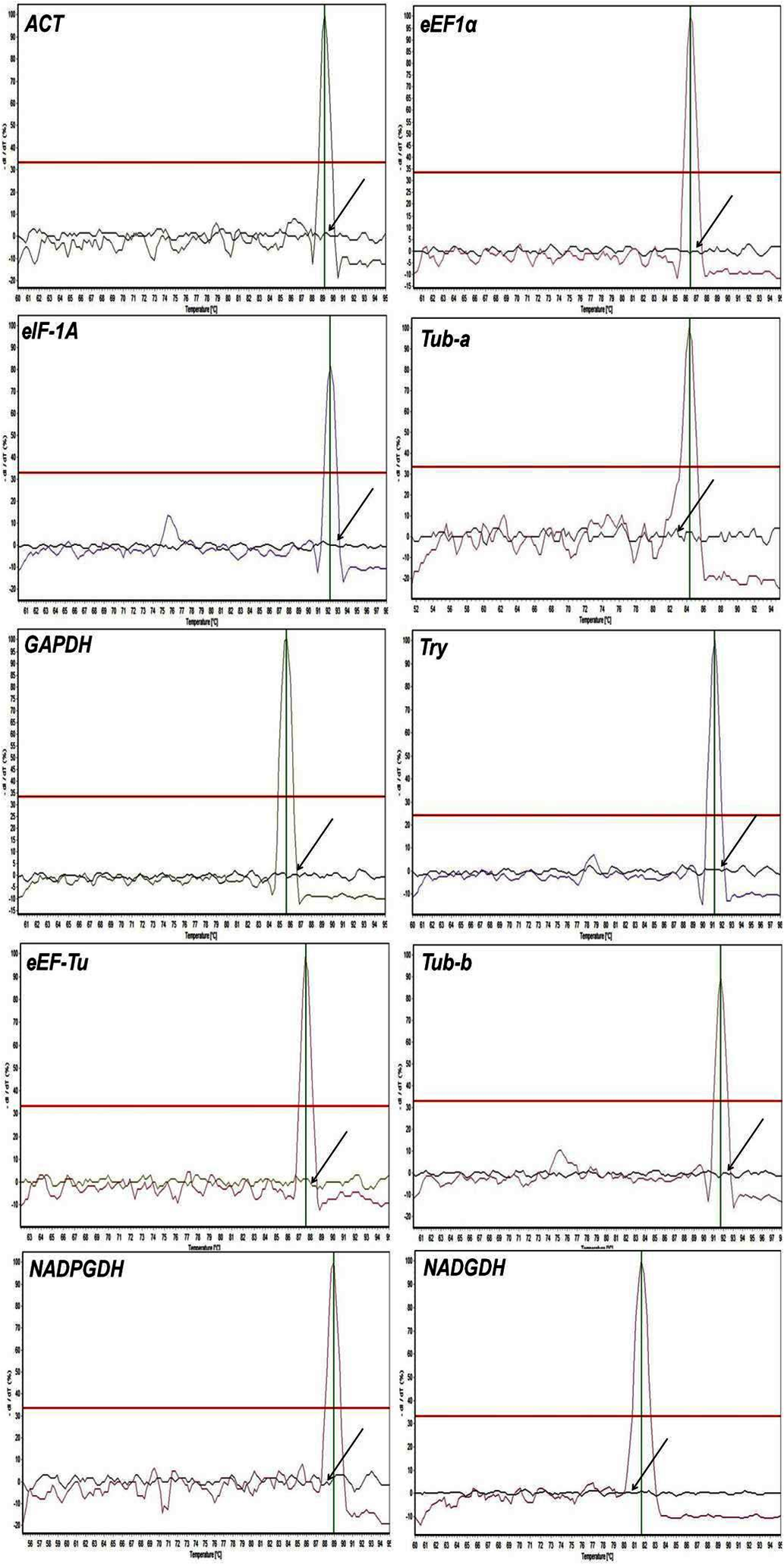

Supplement: S2 Fig — A single sharp peak was observed for each gene product. The melting curve for no template control (NTC) is indicated by arrow. (TIF) [file pone.0179454.s002.tif]

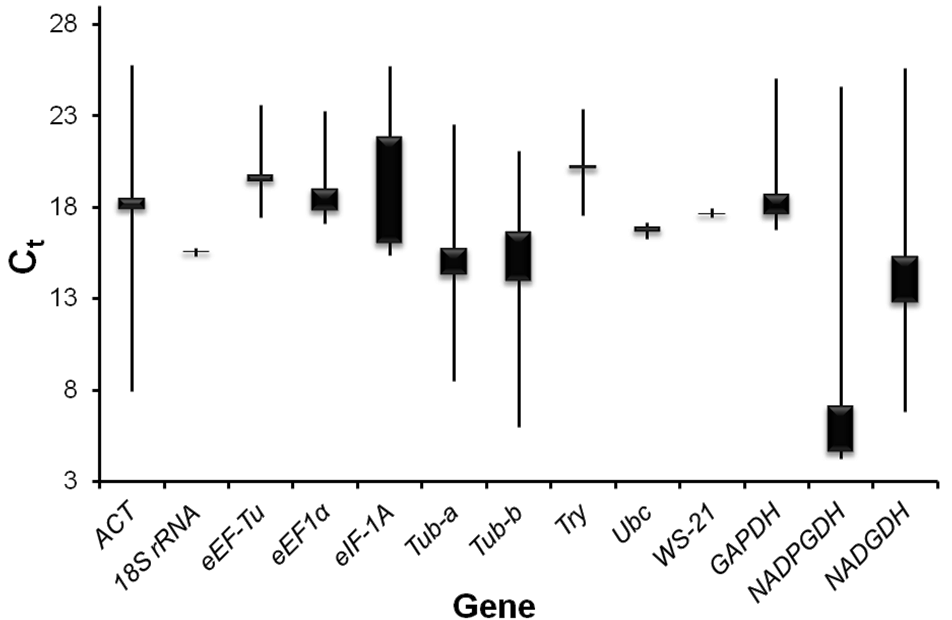

Supplement: S3 Fig — (TIF) [file pone.0179454.s003.tif]
