# Supplementary material for: Selection of reference genes for quantitative real-time RT-PCR assays in different morphological forms of dimorphic zygomycetous fungus Benjaminiella poitrasii
Source: PLoS One. 2017 Jun 9;12(6):e0179454. doi: 10.1371/journal.pone.0179454 (PMC5466344; doi:10.1371/journal.pone.0179454)
Supplement: S1 Table — (DOCX) [file pone.0179454.s004.docx]

**S1 Table.** Analysis of CP data of candidate reference genes by BestKeeper s/w: **during vegetative stage (Yeast-hypha dimorphism)**

|  | ***18S***  ***rRNA*** | ***eEF***  ***1α*** | ***eIF- 1A*** | ***Tub-a*** | ***Tub-b*** | ***Try*** | ***Ubc*** | ***WS-21*** | ***GAPDH*** | ***ACT*** | ***eEF-Tu*** | ***NADPGDH*** | ***NAD***  ***GDH*** |
| --- | --- | --- | --- | --- | --- | --- | --- | --- | --- | --- | --- | --- | --- |
| **N** | **6** | **6** | **6** | **6** | **6** | **6** | **6** | **6** | **6** | **6** | **6** | **6** | **6** |
| **geo Mean [CP]** | 15.55 | 17.37 | 15.61 | 15.28 | 15.48 | 20.37 | 17.00 | 17.66 | 17.59 | 13.57 | 18.87 | 8.52 | 9.67 |
| **ar Mean [CP]** | 15.55 | 17.37 | 15.62 | 15.28 | 15.61 | 20.38 | 17.00 | 17.66 | 17.60 | 14.49 | 18.91 | 9.67 | 10.65 |
| **min**  **[CP]** | 15.47 | 17.08 | 15.34 | 14.78 | 12.90 | 19.92 | 16.92 | 17.62 | 16.83 | 7.89 | 17.41 | 4.25 | 5.23 |
| **max**  **[CP]** | 15.67 | 17.79 | 16.02 | 15.72 | 17.31 | 21.12 | 17.12 | 17.70 | 18.37 | 17.86 | 19.94 | 15.56 | 15.29 |
| **std dev**  **[± CP]** | 0.08 | 0.28 | 0.27 | 1.34 | 1.80 | 0.50 | 0.08 | 0.13 | 0.51 | 4.40 | 1.00 | 4.22 | 4.26 |
| **CV**  **[% CP]** | 0.50 | 1.61 | 1.72 | 2.20 | 11.56 | 2.43 | 0.48 | 0.56 | 2.92 | 30.37 | 5.28 | 43.64 | 39.96 |
| **min**  **[x-fold]** | -1.06 | -1.22 | -1.21 | -1.41 | -5.98 | -1.37 | -1.05 | -1.08 | -1.69 | -51.24 | -2.76 | -19.29 | -21.75 |
| **max**  **[x-fold]** | 1.08 | 1.34 | 1.33 | 1.36 | 3.56 | 1.68 | 1.06 | 1.12 | 1.72 | 19.57 | 2.09 | 131.61 | 49.09 |
| **std dev**  **[± x-fold]** | 1.06 | 1.21 | 1.20 | 2.26 | 3.49 | 1.41 | 1.06 | 1.08 | 1.43 | 21.14 | 2.00 | 18.64 | 19.12 |

n-number of samples analyzed; CP-cross-point value or Ct value; CV-coefficient of variation; geo Mean-geometric mean; ar Mean- arithmetic mean; std dev- standard deviation.
