# Supplementary material for: Selection of reference genes for quantitative real-time RT-PCR assays in different morphological forms of dimorphic zygomycetous fungus Benjaminiella poitrasii
Source: PLoS One. 2017 Jun 9;12(6):e0179454. doi: 10.1371/journal.pone.0179454 (PMC5466344; doi:10.1371/journal.pone.0179454)
Supplement: S2 Table — (DOCX) [file pone.0179454.s005.docx]

**S2 Table.** Analysis of CP data of candidate reference genes by BestKeeper s/w: **during asexual stage (Sporangiospores formation)**

|  | ***18S***  ***rRNA*** | ***eEF***  ***1α*** | ***eIF- 1A*** | ***Tub-a*** | ***Tub-b*** | ***Try*** | ***Ubc*** | ***WS-21*** | ***GAPDH*** | ***ACT*** | ***eEF-Tu*** | ***NADPGDH*** | ***NAD***  ***GDH*** |
| --- | --- | --- | --- | --- | --- | --- | --- | --- | --- | --- | --- | --- | --- |
| **N** | **3** | **3** | **3** | **3** | **3** | **3** | **3** | **3** | **3** | **3** | **3** | **3** | **3** |
| **geo Mean [CP]** | 15.57 | 17.79 | 23.21 | 14.17 | 13.89 | 21.57 | 16.51 | 17.62 | 17.92 | 21.94 | 18.55 | 10.01 | 19.07 |
| **ar Mean [CP]** | 15.57 | 17.79 | 23.27 | 14.21 | 14.38 | 21.60 | 16.51 | 17.62 | 17.92 | 22.10 | 18.61 | 10.32 | 19.26 |
| **min**  **[CP]** | 15.51 | 17.24 | 20.96 | 12.69 | 9.78 | 20.12 | 16.47 | 17.42 | 17.44 | 17.93 | 16.69 | 7.21 | 15.78 |
| **max**  **[CP]** | 15.67 | 18.01 | 25.65 | 15.40 | 20.02 | 23.33 | 16.53 | 17.90 | 18.30 | 25.77 | 20.96 | 13.20 | 22.15 |
| **std dev**  **[± CP]** | 0.04 | 0.22 | 1.41 | 1.02 | 3.48 | 0.98 | 0.02 | 0.19 | 0.93 | 2.32 | 1.32 | 2.07 | 2.32 |
| **CV**  **[% CP]** | 0.25 | 1.26 | 6.04 | 7.18 | 24.20 | 4.52 | 0.13 | 0.35 | 1.30 | 10.49 | 7.11 | 20.08 | 12.04 |
| **min**  **[x-fold]** | -1.04 | -1.46 | -4.77 | -2.79 | -17.24 | -2.73 | -1.03 | -1.15 | -1.39 | -16.07 | -3.62 | -6.97 | -9.78 |
| **max**  **[x-fold]** | 1.07 | 1.17 | 5.41 | 2.35 | 70.17 | 3.39 | 1.02 | 1.22 | 1.31 | 14.26 | 5.33 | 9.12 | 8.46 |
| **std dev**  **[± x-fold]** | 1.03 | 1.17 | 2.65 | 2.03 | 11.15 | 1.97 | 1.01 | 1.14 | 1.88 | 4.99 | 2.50 | 4.20 | 4.99 |

N-number of samples analyzed; CP-cross-point value or Ct value; CV-coefficient of variation; geo Mean-geometric mean; ar Mean- arithmetic mean; std dev- standard deviation.
