# Supplementary material for: Selection of reference genes for quantitative real-time RT-PCR assays in different morphological forms of dimorphic zygomycetous fungus Benjaminiella poitrasii
Source: PLoS One. 2017 Jun 9;12(6):e0179454. doi: 10.1371/journal.pone.0179454 (PMC5466344; doi:10.1371/journal.pone.0179454)
Supplement: S3 Table — (DOCX) [file pone.0179454.s006.docx]

**S3 Table.** Analysis of CP data of candidate reference genes by BestKeeper s/w: **during sexual stage (zygospores formation)**

|  | ***18S***  ***rRNA*** | ***eEF***  ***1α*** | ***eIF- 1A*** | ***Tub-a*** | ***Tub-b*** | ***Try*** | ***Ubc*** | ***WS-21*** | ***GAPDH*** | ***ACT*** | ***eEF-Tu*** | ***NADP***  ***GDH*** | ***NAD***  ***GDH*** |
| --- | --- | --- | --- | --- | --- | --- | --- | --- | --- | --- | --- | --- | --- |
| **N** | **3** | **3** | **3** | **3** | **3** | **3** | **3** | **3** | **3** | **3** | **3** | **3** | **3** |
| **geo Mean [CP]** | 15.54 | 20.47 | 23.38 | 13.08 | 11.48 | 19.22 | 16.59 | 17.60 | 19.54 | 17.45 | 20.83 | 10.76 | 20.61 |
| **ar Mean [CP]** | 15.54 | 20.54 | 23.42 | 13.92 | 12.89 | 19.27 | 16.59 | 17.60 | 19.72 | 17.55 | 20.91 | 11.20 | 20.97 |
| **min**  **[CP]** | 15.31 | 18.07 | 21.69 | 8.47 | 5.94 | 17.54 | 16.24 | 17.43 | 16.76 | 14.94 | 17.98 | 7.23 | 16.13 |
| **max [CP]** | 15.76 | 23.22 | 25.70 | 22.52 | 21.04 | 21.95 | 16.95 | 17.82 | 25.04 | 20.50 | 23.57 | 14.56 | 25.61 |
| **std dev [± CP]** | 0.13 | 1.41 | 1.15 | 4.44 | 5.28 | 1.19 | 0.08 | 0.20 | 2.22 | 1.68 | 1.56 | 2.65 | 3.23 |
| **CV**  **[% CP]** | 1.14 | 6.87 | 4.91 | 31.87 | 40.96 | 6.16 | 1.02 | 1.18 | 11.24 | 9.60 | 7.47 | 23.64 | 15.40 |
| **min**  **[x-fold]** | -1.17 | -5.26 | -3.23 | -24.47 | -46.58 | -3.20 | -1.07 | -1.22 | -6.89 | -5.68 | -7.21 | -11.52 | -22.25 |
| **max**  **[x-fold]** | 1.17 | 6.75 | 4.98 | 693.30 | 753.94 | 6.63 | 1.09 | 1.19 | 45.00 | 8.31 | 6.66 | 13.97 | 32.09 |
| **std dev [± x-fold]** | 1.10 | 2.66 | 2.22 | 21.65 | 38.87 | 2.28 | 1.05 | 1.17 | 4.65 | 3.21 | 2.95 | 6.27 | 9.38 |

N-number of samples analyzed; CP-cross-point value or Ct value; CV-coefficient of variation; geo Mean-geometric mean; ar Mean- arithmetic mean; std dev- standard deviation.
